# Validity, reliability, and user perspectives of the newly developed joint angle measurement system: a preliminary study

**DOI:** 10.1038/s41598-025-25640-x

**Published:** 2025-11-24

**Authors:** Taiki Yoshida, Shintaro Uehara, Asuka Hirano, Shota Itoh, Yohei Otaka

**Affiliations:** 1 Faculty of Rehabilitation, Fujita Health University School of Health Sciences, 1-98 Dengakugakubo, Kutsukakecho, Toyoake, Aichi 470-1101 Japan; 2 Department of Rehabilitation, Fujita Health University Hospital, Toyoake, Aichi Japan; 3 Department of Rehabilitation Medicine, Fujita Health University School of Medicine, 1-98 Dengakugakubo, Kutsukakecho, Toyoake, Aichi 470-1101 Japan

**Keywords:** Inertial measurement unit, Joint angle, Validity, Reliability, Usability, Satisfaction, Rehabilitation, Health care, Medical research

## Abstract

We aimed to evaluate the applicability of a newly developed joint angle measurement system comprising six-axis inertial measurement unit sensors and tablet-based application for estimating joint angles from angular velocity and acceleration data. The application calculated orientation angles from single sensor data, with relative angles calculated using multiple sensors. In experiment 1, validity and reliability were examined using a test device. In experiment 2, static angles of five joints were calculated in four healthy participants using attached sensors and compared with universal goniometer values. In experiment 3, usability and satisfaction were evaluated using the System Usability Scale (SUS) and Quebec User Evaluation of Satisfaction with Assistive Technology (QUEST)-like scale. In experiment 1, mean difference and root mean squared error (RMSE) between the developed system and test device were < 0.2° and < 1.0°, respectively, across all axes. In experiment 2, when data from all joints were pooled, mean difference and RMSE were 0.2° and 3.8°, respectively. Mean difference and RMSE across each joint were < 5°, indicating the system is comparable to universal goniometer. In experiment 3, median SUS and QUEST-like scores were 73.8 and 4.0, respectively, indicating good usability and satisfaction. The developed system has high accuracy and sufficient validity for human joint angles, with good usability and satisfaction.

**Supplementary Information:**

The online version contains supplementary material available at 10.1038/s41598-025-25640-x.

## Introduction

Identifying the differences between normal and pathological movements to understand the characteristics of impairments is essential for planning rehabilitation exercise and evaluating its effectiveness. Therefore, rehabilitation professionals often use kinematic assessments such as joint angle measurement. Three-dimensional (3D) motion analysis systems using optical markers are commonly used for kinematic measurements, and they have shown good inter-rater variability and allow for the tracking of joint angle changes during active movements^1^. However, this method requires multiple infrared cameras, thus limiting its use to the laboratory^2,3^. Consequently, the measurement of patients’ activities in real-life and clinical settings, such as hospital wards, homes, and outdoor environments, appears impractical. Therefore, there is a need to develop simpler measurement methods as alternatives to 3D motion analysis systems for kinematic measurements in real-life settings.

In recent years, inertial measurement unit (IMU) sensors have gained attention in the field of rehabilitation as a convenient method for measuring joint angles^4–6^, potentially providing a solution to the limitations of 3D motion analysis systems. IMU sensors typically comprise accelerometers, gyroscopes, etc. to estimate posture and joint angles. Previous studies have reported the reliability and validity of IMU sensors for the measurement of the joint angles in the neck^7^, shoulder^8–11^, elbow^10–13^, wrist^10,11^, hip^14,15^, knee^16,17^, and trunk^18,19^. In addition, similar to the 3D motion analysis system using optical markers, IMU-based measurements allow the continuous tracking of joint movements during active motion^20^. Therefore, IMU sensors can become a better alternative method for measuring human joint angles in rehabilitation settings. However, most studies on the applicability of IMU sensors in rehabilitation settings were performed at the laboratory level, and there is limited research on their feasibility for daily clinical use in real-world settings. One major barrier is the absence of user-friendly, integrated systems that can capture and analyze upper and lower limb joints and trunk angles within a single platform^21^; besides, applications optimized for rehabilitation workflows are lacking. To overcome these limitations, we developed a novel IMU-based joint angle measurement system, featuring a tablet-based application with an interface that enables easy, rapid measurement, automatic data storage, and streamlined analysis of multiple joint angles. This system was specifically designed for practical clinical use, requiring minimal setup, allowing independent operation by rehabilitation professionals without technical expertise, and enabling seamless integration into routine rehabilitation sessions.

In this preliminary study, we aimed to evaluate the clinical feasibility of the newly developed joint angle measurement system by investigating the validity and reliability of the calculated angles, usability of the system, and user satisfaction. Specifically, in experiment 1, we investigated whether the developed system could accurately measure orientation angles. In experiment 2, as a preliminary evaluation toward developing a system capable of capturing dynamic human movements, we calculated joint angles in static positions for five joints in the upper, lower limbs, and trunk using the IMU sensors attached to the human body to examine the bias in the angles from those measured by the universal goniometer. In experiment 3, we evaluated the usability and user satisfaction of the newly developed measurement system using questionnaires in rehabilitation professionals.

## Methods

### Newly developed joint angle measurement system

**Fig. 1:**
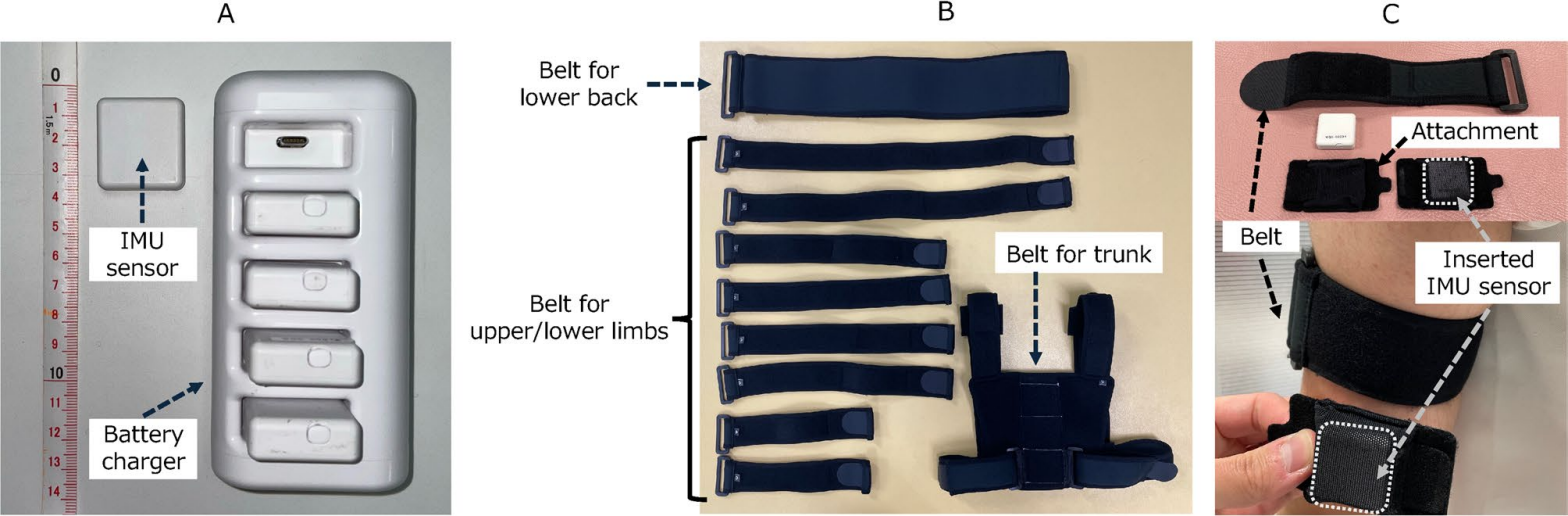
(**A**) Overview of the developed angle measurement system. The system consists of IMU sensors, a tablet-based application, and a battery charger. (**B**) The specialized belts for the upper, lower limbs, lower back, and trunk were used in the study. The surface has Velcro fasteners for attachment. (**C**) The specialized attachment used in the study. The IMU sensor was inserted in the attachment, which was secured to the belt worn on the body with the Velcro fastener. IMU: inertial measurement unit.

We developed a joint‑angle measurement system comprising custom six‑axis IMU modules (each integrating a tri-axial accelerometer and a tri-axial gyroscope), an angle‑calculation application running on an iPad (3rd generation) [Apple Inc., Cupertino, CA, USA], and a dedicated charger/alignment dock (Fig. 1). The IMU module (28.5 × 30.0 × 11.4 mm) measurement ranges were ± 8 g for acceleration and ± 500°/s for angular velocity, and its noise densities were 180 µg/√Hz^−1^ and 0.007° s^−1^ √Hz^−1^, respectively. Sensor data were sampled at 100 Hz and downsampled to 20 Hz when transmitted to the tablet via Bluetooth Low Energy to avoid excessive data volume and analysis load. Previous studies have shown that 20 Hz can capture essential movement characteristics in the daily activities of rehabilitation patients^22,23^; therefore, we adopted 20 Hz to reduce battery consumption and facilitate clinical use. The tablet is equipped with an internal real-time clock (RTC). Upon reception, the tablet assigns an RTC-based timestamp to every data, enabling synchronization of data streams from multiple sensors on a common timeline. Within each IMU, the Kalman filter fuses tri-axial acceleration and angular‑velocity signals to obtain orientation angles in the sensor frame. Each IMU sensor underwent a six-face static calibration (3 s per face) in the charging dock to compute scale and bias parameters and establish a common world frame. Pitch and roll were further corrected online using the gravity vector. After mounting, the participant briefly (< 1 s) held a predefined neutral posture; the orientation during this pause, alongside the predefined attachment site and sensor orientation registered in the application, was used to map the world frame to the anatomical reference frame of each segment. This calibration procedure was repeated whenever a sensor was re-mounted. Joint angles were calculated as the relative orientation between two sensors mounted on adjacent segments.

Regarding the attachment method of the sensors, surgical tape was initially used to directly affix the sensors to the skin. However, displacement of the sensors occurred during measurement (see Supplemental data 1). To address this, we developed specialized sensor attachments and a set of adjustable belts to secure them (Fig. 1). For the upper and lower limbs and lower back, we used adjustable elastic nylon belts of different lengths and widths; for the trunk, we used a combined belt system comprising chest and shoulder straps. This belt is adjustable with Velcro fasteners. Each IMU sensor was placed inside a fabric pouch attachment with a Velcro fastener on its back, and this pouch was secured to the belt. This design allowed for stable fixation while accommodating different body sizes and reducing measurement differences compared to securing the sensors using tape.

### Experiment 1:　accuracy of the calculated angles

**Fig. 2:**
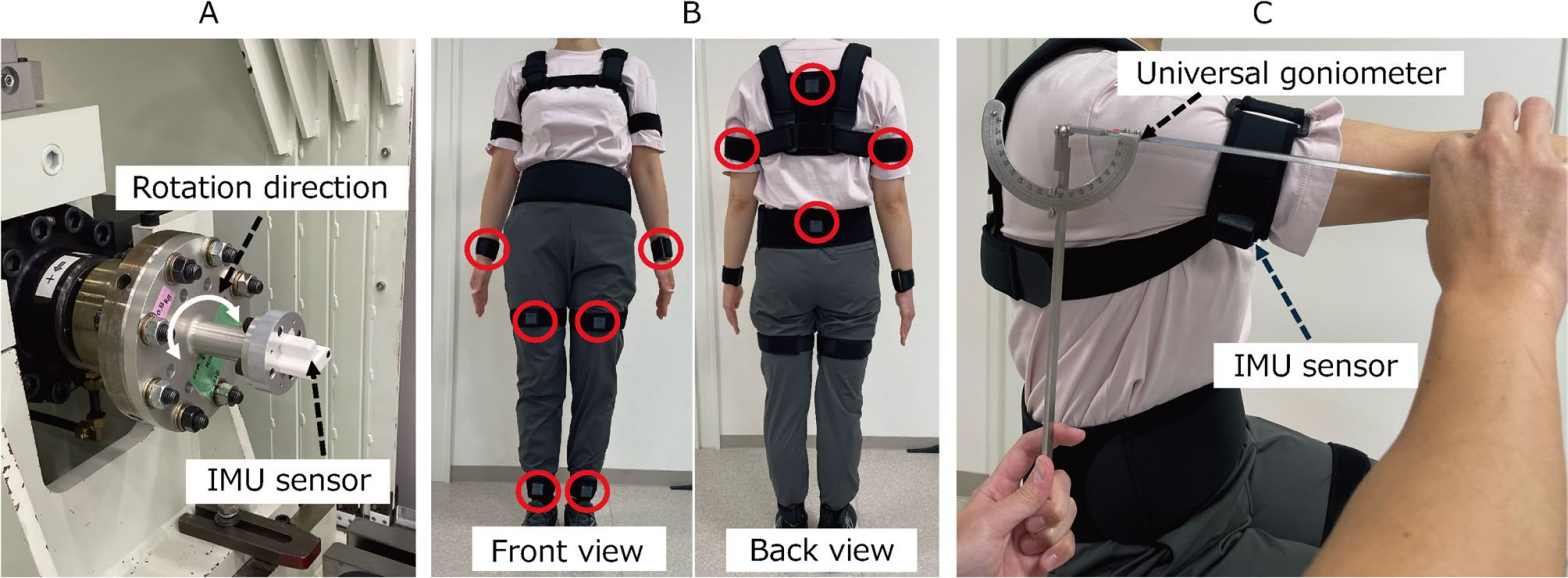
(**A**) Test device used in the Experiment (1) The test device repeatedly rotates back and forth, with a baseline of 0°, reaching 42.5° on each side. (**B**) The position of the IMU sensors attached to the human body. Red circles indicate sensor attachment sites. (**C**) An example view of angle measurement in the Experiment (2) The joint angle was measured simultaneously using the developed system and universal goniometer. IMU: inertial measurement unit.

To verify the accuracy of the calculated angles obtained using the developed measurement system, we used a test device that repetitively moved at specified angles (Fig. 2). The device performs repetitive rotating movements from a neutral position of 0°, reaching up to ± 42.5° to each side. As it approaches the maximum (+ 42.5°) or minimum angle (-42.5°), the rotational speed gradually decreases. One complete cycle of movement (from + 42.5° to -42.5° and back) takes approximately 8 s. The IMU sensor was secured to ensure the alignment of its rotational axis aligned with the roll, pitch, and yaw axes, and each axis underwent 10 cycles of back-and-forth rotation. The timing of the start of movement of the two measurement methods was visually identified and synchronized during offline analysis by checking the timing of the two datasets.

First, to investigate the validity of the calculated angles, we analyzed the bias in the angles obtained over time from the test device and measurement system during the 10 cycles of rotation using Bland–Altman analysis. Next, to confirm the reliability of the calculated angles, we focused on the angles obtained at the transition points of the rotating directions (i.e., the maximum and minimum angles) of the test device. Two sets of 10 cycles of rotation were measured (first and second sets), and the differences in the 10 maximum and minimum angles between the first and second sets were evaluated using Bland–Altman analysis. We calculated angle differences, standard deviation (SD), standard error of measurement (SEM), root mean squared error (RMSE), intraclass correlation coefficient (ICC), Cohen’s d, and 95% confidence interval (95% CI) of the bias, as well as the Upper Limits of Agreement (ULoA) and its 95% CI and the Lower Limits of Agreement (LLoA) and its 95% CI. The presence of fixed bias was confirmed when the 95% CI of the differences between the two measurement methods or two sessions did not include zero. Proportional bias was tested using a linear regression analysis and confirmed when the regression coefficient was not equal to zero (*p* < 0.05). R (version 4.4.2) was used for data analysis.

### Experiment 2: accuracy of the system for human joint angle measurement

Experiment 2 was conducted with the approval of the Ethics Review Committee of Fujita Health University (approval number: HM21-029) and in accordance with the Declaration of Helsinki of 1964, as revised in 2013. Before conducting the measurements, the researchers provided the participants with an oral explanation and written materials detailing the study, and written consent was obtained from each participant prior to the experiments.

Experiment 2 aimed to verify the accuracy of the calculated angles when measuring the human joint angle at several static positions by comparing them with those measured using the universal goniometer, a commonly used joint angle measurement device in clinical settings. Four healthy volunteers (two females) with a mean age (SD) of 29.0 (4.0) years participated in the experiment. The inclusion criterion was the absence of any past medical history or disorders that could limit the range of motion of the target joints. The IMU sensors were attached to the following locations with a specialized attachment (Fig. 1): the posterior part of the mid-upper arm, the dorsal part of the distal forearm, the back at the level of the first thoracic vertebra (spinous process) and at the level between the spinous processes of the fourth and fifth lumbar vertebrae (intersection of the Jacoby’s line and the spine), the anterior part of the mid-thigh, and the anterior part of the distal lower leg.

Participants held the arbitrary position at 10 different angles for each of the five joint movements: shoulder flexion, elbow flexion, hip flexion, knee flexion, and trunk flexion. The angle of the five joint movements were calculated using the following sensor combinations: shoulder flexion, sensors on the posterior mid-upper arm and the back at the first thoracic vertebra; elbow flexion, sensors on the posterior mid-upper arm and dorsal distal forearm; hip flexion, sensors between the fourth and fifth lumbar vertebrae and anterior mid-thigh; knee flexion, sensors on the anterior mid-thigh and anterior distal lower leg; and trunk flexion, sensors on the back at the level of the first thoracic vertebra and at the level between the spinous processes of the fourth and fifth lumbar vertebrae. To compare the calculated angles of the measurement system with those measured using the universal goniometer, two raters—an occupational therapist with 15 years of clinical experience and a physical therapist with 21 years of clinical experience—measured the joint angles with the universal goniometer while simultaneously measuring the static joint angles with the measurement system. Both raters checked the angle measured using the universal goniometer and reached a consensus on each measurement value (1° increment).

The analysis was first performed by integrating all measured ten angles of the five joints from all four participants; afterward, the bias of angles between the measurement system and universal goniometer were examined using Bland–Altman analysis. Subsequently, the same analysis was performed separately for the angles of each joint. We calculated the difference of angles, SD, RMSE, Cohen’s d, 95% CI of the bias, ULoA and its 95% CI, and LLoA and its 95% CI. The presence of a fixed bias was confirmed when the 95% CI of the differences between the two measurement methods did not include zero. Proportional bias was tested using a linear regression analysis and confirmed when the regression coefficient was not equal to zero (*p* < 0.05). R (version 4.4.2) was used for the analysis.

### Experiment 3: evaluation of usability and user satisfaction

Experiment 3 was conducted following the same procedure as that of Experiment 2, with the approval of the Ethical Review Committee and consent of the participants. Because the developed joint angle measurement system was intended for use as a clinical assessment tool, the end users were assumed to be rehabilitation professionals. Therefore, rehabilitation professionals were recruited as participants for this experiment. Sixteen rehabilitation professionals from the Department of Rehabilitation at Fujita Health University Hospital participated in this experiment: twelve physical therapists (five female; mean [SD] years of clinical experience, 4.2 [5.0]) and four occupational therapists (one female; mean [SD] years of clinical experience, 7.5 [4.7]). The inclusion criterion was at least 1 year of clinical experience as a physical or occupational therapist. After a detailed explanation of how to use the developed measurement system, participants were asked to perform a set of standardized tasks using the developed system, including sensor attachment, operation of the tablet application, and data retrieval. Participants received approximately 60 min of hands-on training to familiarize themselves with the system, followed by opportunities to use it over the subsequent 2 weeks. The number of system uses during this period was recorded. Following this session, participants completed the System Usability Scale (SUS)^24^ and a modified version of the Quebec User Evaluation of Satisfaction with Assistive Technology (QUEST)^25^ questionnaires to evaluate the usability of and satisfaction with the system. The SUS is a reliable measure for assessing the usability of various products and services. It consists of 10 items scored on a scale of 0 to 100, with participants rating each item on a five-point Likert scale, ranging from “strongly agree” to “strongly disagree.” Higher scores indicate greater usability of the system. An SUS score below 50 indicates that users rated the system as “OK,” whereas scores of 70 or higher are considered “good,” and scores above 85 are deemed “excellent.” The QUEST is a reliable measure for evaluating user satisfaction with assistive technology. It consists of items regarding assistive devices and services. In the present study, we only used the items regarding assistive devices to better suit the evaluation of the developed system: size (height, length, and width), weight, ease of adjustment (installation and adjustment of components), safety, durability, ease of use, comfort, and effectiveness. Each aspect was rated on a five-point Likert scale, comprising “1: Not satisfied at all”, “2: Not very satisfied”, “3: more or less satisfied”, “4: Quite satisfied”, and “5: very satisfied.” The mean score across all items was calculated as the overall satisfaction score for the system, and this score was interpreted with higher scores reflecting higher levels of satisfaction.

## Results

### Experiment 1

**Fig. 3:**
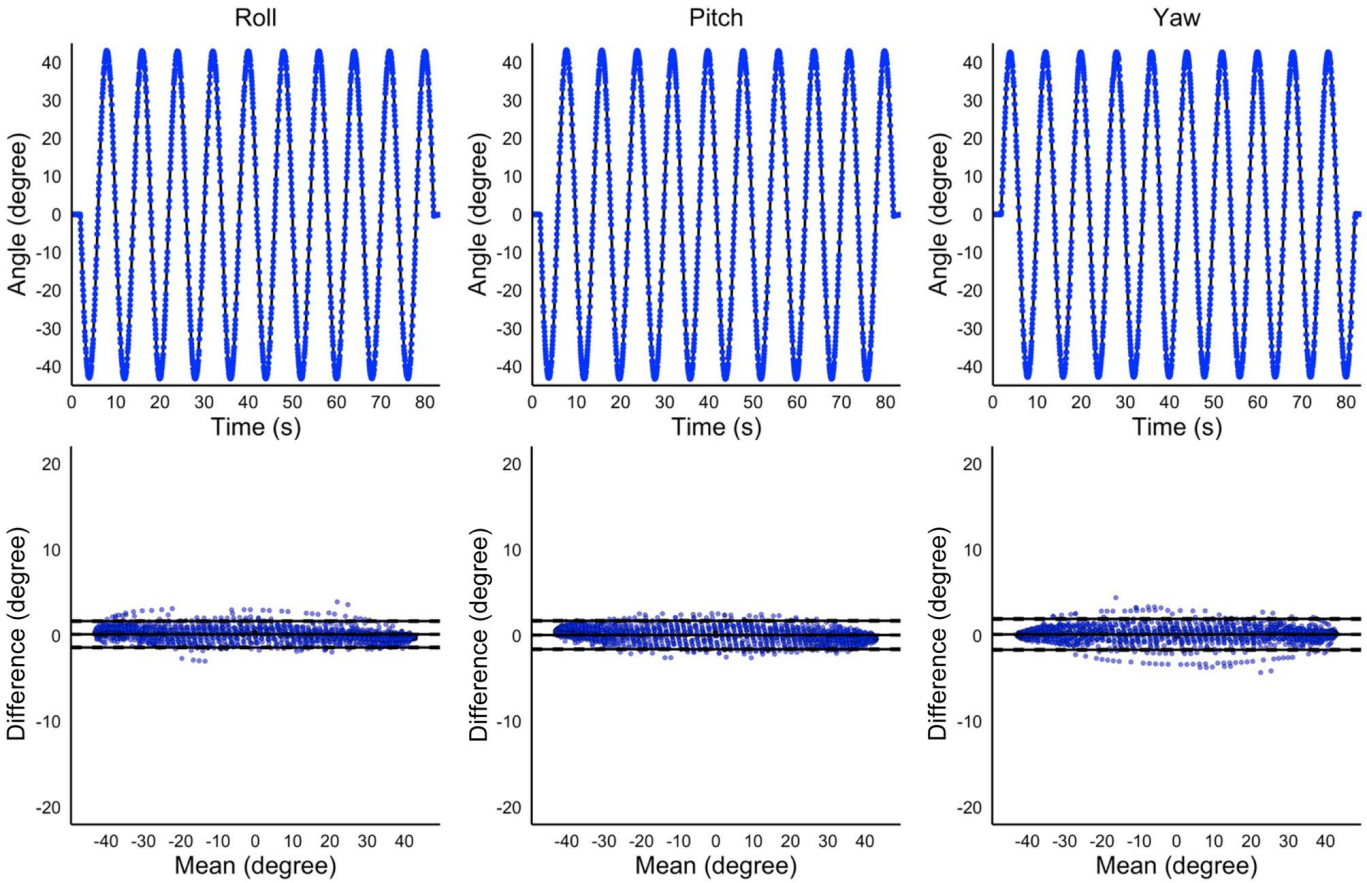
The mean differences of the measured joint angles between the developed system and test device. The top three panels represent the time-series of angles of the test device (solid lines) and output angles measured using the developed system (dotted lines). The X and Y axes show the time and the measured angle, respectively. The bottom three panels represent the mean differences in the measured angle between the two measurement methods using the Bland–Altman plots. Each plot represents the angle of the test device minus the angle calculated by the developed system. X and Y axes show the mean value of the two measurement methods and the mean difference between them, respectively. The middle solid line represents the mean difference; the top and bottom solid lines show the Limits of Agreement. The dotted lines show the 95% confidence intervals of the mean difference and the Limits of Agreement.

The Bland–Altman plot of time-series data obtained using the measurement system and test device (i.e., validity relative to the angles obtained from the test device) revealed a statistically significant fixed bias for the Roll and Yaw axes (*p* < 0.001) and proportional bias in the Pitch and Roll axes (*p* < 0.001). However, the mean differences were < 0.2°, RMSE were < 1.0°, and the ULoA and LLoA were < 2° for all axes (Fig. 3; Table 1). In addition, the Bland–Altman plot of the maximum and minimum angles between the first and second sets of 10 cycles of rotating movements obtained using the measurement system (i.e., reliability of the calculated angles) revealed a statistically significant fixed bias in the maximum Pitch and minimum Roll, Pitch, and Yaw angles (*p* = 0.023, *p* = 0.039, *p* = 0.028, and *p* = 0.013, respectively) and no proportional bias was observed in any of the angles. The mean differences were < 0.1° for all axes, with their ULoA and LLoA < 1° (Table 2). These results suggest that, despite the presence of statistically significant fixed and proportional biases in some axes, the magnitude of these biases was minimal from a clinical perspective. Therefore, the calculated angles can be considered valid and reliable.

**Table 1:** Bias of the time-series angles between those calculated using the developed system and test device.

	Roll	Pitch	Yaw
Fixed bias			
Mean difference	0.14	0.05	0.13
SD	0.78	0.84	0.91
RMSE	0.79	0.84	0.92
95% CI	0.10–0.18	0.01–0.09	0.08–0.17
Upper LoA	1.67	1.71	1.93
95% CI	1.59–1.75	1.62–1.80	1.83–2.03
Lower LoA	-1.38	-1.60	-1.67
95% CI	-1.47–1.30	-1.69–-1.51	-1.76–-1.57
p-value	< 0.001	0.007	< 0.001
Cohen’s d	0.183	0.065	0.144
Proportional bias			
r	-0.008	-0.009	< 0.001
p-value	< 0.001	< 0.001	0.366

SD: standard deviation; RMSE: root mean squared error; LoA: Limits of Agreement; CI: confidence interval.

**Table 2:** Bias of the maximum and minimum angle between the first and second sets of 10 cycles of rotational motion calculated using the measurement system.

	Maximum angles			Minimum angles		
	Roll	Pitch	Yaw	Roll	Pitch	Yaw
Fixed bias						
Mean difference	0.01	0.02	0.01	0.01	0.01	0.02
SD	0.02	0.02	0.04	0.01	0.01	0.02
SEM	0.00	0.00	0.01	0.00	0.00	0.00
95% CI	0.00–0.02	0.00–0.04	-0.01–0.04	0.00–0.02	0.00–0.03	0.00–0.04
Upper LoA	0.06	0.07	0.09	0.05	0.05	0.07
95% CI	0.03–0.08	0.04–0.10	0.05–0.14	0.02–0.07	0.02–0.07	0.04–0.11
Lower LoA	-0.03	-0.02	-0.06	-0.02	-0.02	-0.02
95% CI	-0.06–0.01	-0.06–0.00	-0.10–-0.01	-0.04–0.00	-0.04–0.00	-0.06–0.00
ICC^1,2^	0.957	0.944	0.315	0.976	0.975	0.612
p-value	0.195	0.023	0.190	0.039	0.028	0.013
Proportional bias						
r	-0.031	-0.065	-0.672	0.013	-0.250	0.504
p-value	0.850	0.573	0.219	0.970	0.485	0.136

SD: standard deviation; SEM: standard error of measurement; LoA: Limits of Agreement; CI: Confidence interval; ICC: intraclass correlation coefficient.

### Experiment 2

**Fig. 4:**
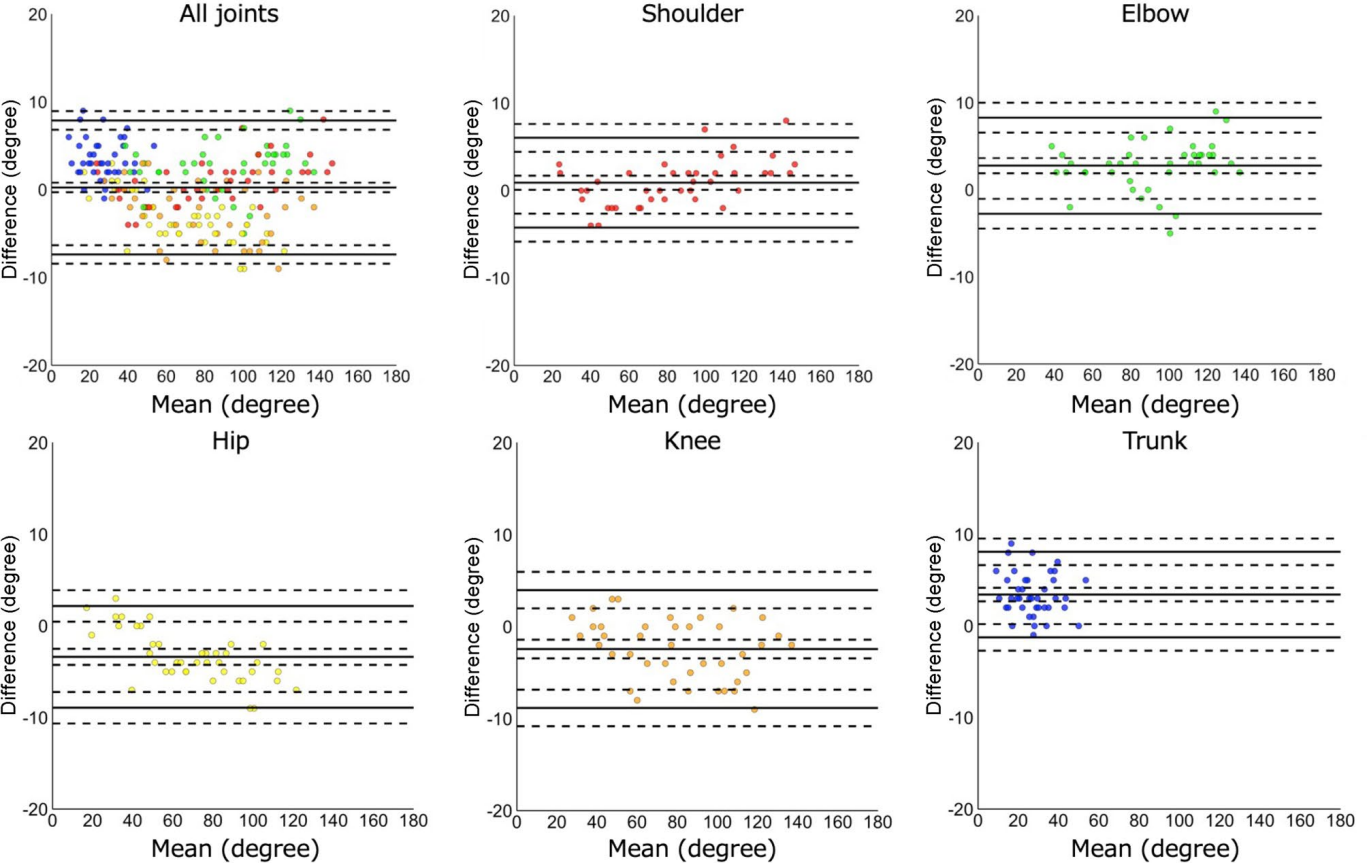
The mean difference of the measured joint angles between the developed system and universal goniometer. Each plot represents the angle measured by the universal goniometer minus the angle calculated by the developed system. The top left panel represents the mean differences in the measured angles between the two methods using the Bland–Altman plots, with all joint movement data integrated. The other five panels represent the mean differences for each joint: shoulder (top center), elbow (top right), hip (bottom left), knee (bottom center), and trunk (bottom right). X and Y axes show the mean value of the two measurement methods and the mean difference between them, respectively. The middle solid line represents the mean differences; the top and bottom solid lines show the Limits of Agreement. The dotted lines show the 95% confidence intervals of the mean differences and the Limits of Agreement.

In the Bland–Altman plot comparing the developed system with the universal goniometer using the integrated dataset comprising ten angles of five joints from four participants, a statistically significant proportional bias was observed (*p* = 0.016); however, no fixed bias was confirmed. The mean difference was 0.2°, RMSE was 3.8°, the ULoA was 7.8°, and the LLoA was-7.3°. Analysis of each individual joint revealed a statistically significant fixed bias in all joints (*p* < 0.05), and a proportional bias was observed in the shoulder, hip, and knee joints (*p* < 0.05). However, even the largest mean difference was 3.4° in the trunk, and the largest ULoA and LLoA were 3.9° and − 8.8°, respectively, in the knee joint. This result indicates minimal differences when considering potential effects in the clinical setting. These results suggest that, despite the presence of some fixed or proportional biases, the joint angles calculated using the developed measurement system have acceptable validity relative to those obtained using the universal goniometer (Fig. 4; Table 3).

**Table 3:** Bias of the joint angles between those measured using the developed system and universal goniometer.

	All joints	Shoulder	Elbow	Hip	Knee	Trunk
Fixed bias						
Mean difference	0.2	0.9	2.7	-3.3	-2.4	3.4
SD	3.8	2.6	2.8	2.8	3.2	3.4
RMSE	3.8	2.7	3.9	4.3	4.0	4.1
95% CI	-0.2–0.8	0.0–1.7	1.8–3.6	-4.2–-2.4	-3.4–-1.3	2.6–4.1
Upper LoA	7.8	6.0	8.2	2.1	3.9	8.1
95% CI	6.7–9.0	4.1–7.8	6.3–10.2	0.1–4.1	1.6–6.2	6.4–9.7
Lower LoA	-7.3	-4.2	-2.7	-8.9	-8.8	-1.2
95% CI	-8.5–-6.1	-6.0–-2.8	-4.7–-0.7	-10.9–-6.9	-11.1–-6.5	-2.9–0.4
p-value	0.346	0.035	< 0.001	< 0.001	< 0.001	< 0.001
Cohen’s d	0.066	0.344	0.986	1.191	0.741	1.436
Proportional bias						
r	-0.018	0.039	0.022	-0.072	-0.034	-0.030
p-value	0.016	< 0.001	0.160	< 0.001	0.046	0.401

SD: standard deviation; RMSE: root mean squared error; LoA: Limits of Agreement; CI: Confidence interval.

### Experiment 3

The median number of uses during the trial period was 3.6 (IQR 1.7–5.0). Although there were some differences between individuals, the median SUS score was 73.8 (IQR: 62.5–78.1), demonstrating the usability of the system as “good.” The median score for the QUEST-like scale was 4.0 (IQR: 4.0–4.1), indicating that the participants were “quite satisfied” with the system. Further analysis of the individual items revealed that the median score for all items was above “3.0: more or less satisfied.” These results on user perspectives demonstrate no significant defects in the system (Table 4)༎.

**Table 4:** Scores of usability and user satisfaction of the developed system.

Participant	1	2	3	4	5	6	7	8	9	10	11	12	13	14	15	16	Median
Number of uses	5	3	6	5	5	2	5	5	5	1	1	1	1	2	5	5	3.6
SUS	77.5	72.5	92.5	77.5	72.5	55.0	67.5	55.0	55.0	75.0	55.0	82.5	77.5	100.0	65.0	80.0	73.8
QUEST-like mean of sub-items	4.3	4.0	4.6	4.0	3.6	3.0	3.9	3.8	3.1	4.4	4.0	4.0	4.5	4.9	3.5	4.1	4.0
Size	5	4	5	4	3	4	4	4	4	4	4	3	5	5	4	5	4.0
Weight	5	5	5	4	4	4	4	5	4	5	4	4	5	5	4	5	4.5
Ease of adjustment	4	4	3	4	3	2	4	3	2	3	4	4	5	4	2	3	3.5
Safety	5	5	5	4	5	3	4	5	3	5	4	5	5	5	4	4	5.0
Durability	3	5	5	4	4	3	4	3	3	5	4	3	4	5	3	3	4.0
Ease of use	4	2	5	4	3	2	3	3	2	4	4	5	4	5	3	5	4.0
Comfort	4	3	5	4	3	3	4	3	3	4	4	4	4	5	4	4	4.0
Effectiveness	4	4	4	4	4	3	4	4	4	5	4	4	4	5	4	4	4.0

SUS: System Usability Scale; QUEST-like: Quebec User Evaluation of Satisfaction with Assistive Technology-like questionnaire.

## Discussion

In the present study, for evaluating the clinical feasibility of the newly developed joint angle measurement system consisting of the IMU sensors and tablet-based application, we investigated the validity and reliability of the calculated values, usability, and user satisfaction. The results showed that the developed system can accurately capture angular changes, demonstrating good usability and user satisfaction. These findings indicate that the newly developed measurement system could contribute to clinical practice for measuring joint angles in the upper limbs, lower limbs, and trunk.

In experiment 1, the validity and reliability of the angle measurements obtained from the developed measurement system was assessed using the test device. The results confirmed that the developed measurement system is capable of measuring highly variable and reliable temporal angle data. Regarding the ICC results, the yaw axis showed a relatively low value. This is probably because the between-subject variance of the yaw axis was smaller than that of the other axes. As a result, the difference between the between-subject and within-subject variances was small, thereby leading to the lower ICC^26^ (see Supplemental data 2). Therefore, the low ICC for the yaw axis should be interpreted as a consequence of the limited variability across subjects rather than poor measurement reliability. In experiment 2, we investigated the bias in the measured angles of the measurement system and universal goniometer to confirm the validity of the measurement system in measuring human joint angles. The results showed that the mean difference of the integrated data obtained using the measurement system and universal goniometer was < 1°. Moreover, focusing on individual joints, the bias for all joints was < 5°. These results were comparable to those of previous studies comparing joint angles measured using IMU sensors and universal goniometer^8,9,13,15^. Given that the clinically acceptable error range is suggested to be < 5°^27,28^, the present results indicate that the level of bias is not clinically significant.

However, the mean difference in the measured angles found in experiment 2 was greater than that observed in experiment 1, where the calculated angles were compared with those of the test device. The mean difference in joint angles measured using the developed system and universal goniometer is likely due to biases in the definitions of reference axes for each measure. The axes for the universal goniometer are usually a bone and a reference line in the external environment. In contrast, the axes for the IMU sensor are the body of the device itself. For example, for shoulder flexion, the universal goniometer defines a vertical line to the floor passing through the acromion as the reference axis and the humerus as the other axis^29^. In the developed measurement system, the angle formed between the sensor attached to the back and the sensor attached to the upper arm is defined as shoulder flexion. This mean difference may be a major contributor to the discrepancies in the measured angles, specifically in the following scenarios.

The first scenario is when the muscle mass or amount of adipose tissue at the attachment site changes due to the expansion and contraction of these tissues, along with changes in joint angles^30^. This assumption can be supported by the result of the present study showing that angle bias was larger for lower limb joints and trunk, which have more muscle mass and adipose tissue amount, leading to a greater likelihood of the sensor to shift compared to those for upper limb joints. In addition, this result indicates that caution should be applied to areas with greater muscle mass and adipose tissue amount, and careful consideration is needed to ensure a secure attachment when measuring joint angles using the developed system. These differences may be further minimized by measuring individuals with different body compositions and calculating correction coefficients for each body composition cluster. Such approaches could improve the overall accuracy of the system by adjusting for variations due to individual characteristics.

The second scenario is when the IMU sensor attached to the body moves due to insecure attachment, resulting in misalignment of the sensor’s position. In this study, special attachments were used to secure the sensors to the body to reduce the risk of misalignment (Fig. 2). However, it is still possible that slight sensor misalignment could not be entirely prevented. It has been also demonstrated that when using optical markers attached to the body surface for human motion analysis, misalignment of the markers cannot be eliminated, leaving measuring errors/noise to be removed^31^. Therefore, there is need for a better protocol to secure the IMU sensor to the body. For example, using a rigid plastic attachment rather than a soft fabric pouch may help reduce sensor misalignment and thereby improve measurement accuracy.

In the present study, in addition to evaluating the validity and reliability of the developed system for angle measurement, we evaluated the usability and user satisfaction of the system in experiment 3 from the perspective of practical clinical application. User experience, a comprehensive concept that includes usability, is essential for promoting the clinical adoption of medical devices and enhancing their effectiveness and safety^32,33^. The results demonstrated that even without prior technical expertise, users were able to operate the system after a brief, 60-min hands-on training session, indicating good usability and user satisfaction. The developed system comprises compact IMU sensors, attachments, and a tablet-based application. In contrast to conventional 3D motion analysis systems, which require specialized cameras, software, and expert training, this system offers the potential to significantly reduce time costs associated with motion analysis in clinical settings. Therefore, it can be concluded that the developed system has sufficient potential for clinical application. In the future, it may enable motion analysis in environments that were previously difficult to access, such as hospital wards, homes, and outdoor environments.

This study had several limitations. Regarding the validity of the developed system compared to the universal goniometer, the number of participants and the variety of joints and movements assessed were limited. Individual differences in body composition—such as the amount of muscle mass and adipose tissue, patient age, and types of disabilities, and joint movements such as extension, abduction, and external rotation may have caused biases in the calculated joint angles. Future studies should include participants from diverse populations and assess various joints and movements. Moreover, the present study focused on the validity of joint angles in static positions to evaluate the ability of the system to capture dynamic human movements. To apply the developed system in real-life and clinical settings for motion analysis, future investigations will be needed to validate the joint angles during active movements. Furthermore, future studies should include direct comparisons with other IMU-based systems reported in the literature to comprehensively assess usability and cost-effectiveness, in addition to accuracy. Regarding usability, because all testing was performed by therapists who had at least 1 year of clinical experience, we cannot readily generalize the findings to novice rehabilitation professionals or students.

## Conclusions

The present study evaluated the validity, reliability, usability, and user satisfaction of the newly developed joint angle measurement system consisting of IMU sensors and tablet-based application, confirming its potential for clinical application. The use of the developed system offers the possibility of extending motion analysis from the laboratory settings to more natural and practical environments, such as hospital wards, homes, and outdoor environments. However, further clinical validations are required to establish the applicability of the system.

## Supplementary Information

Below is the link to the electronic supplementary material.

Supplementary Material 1

Supplementary Material 2

Supplementary Material 3

Supplementary Material 4

## Data Availability

The datasets supporting the conclusions of this article are included within the article (and its additional files).
