# Supplementary material for: Validity, reliability, and user perspectives of the newly developed joint angle measurement system: a preliminary study
Source: Sci Rep. 2025 Nov 24;15:41696. doi: 10.1038/s41598-025-25640-x (PMC12644602; doi:10.1038/s41598-025-25640-x)
Supplement: Supplementary file 2 — Supplementary Material 2 [file 41598_2025_25640_MOESM2_ESM.docx]

**Supplemental data: ICC calculation results**

$$ICC\left( 1,2 \right)=\frac{BMS-WMS}{BMS}$$

ICC: Intraclass correlation coefficient; BMS: Between-subject mean square (Between-subject variance); WMS: Within-subject mean square (Within-subject variance)

Maximum angle data

| Cycle | 1^st^ set Pitch | 2^nd^ set Pitch | 1^st^ set Roll | 2^nd^ set Roll | 1^st^ set Yaw | 2^nd^ set Yaw |
| --- | --- | --- | --- | --- | --- | --- |
| 1 | 43.2016 | 43.1836 | 43.0392 | 43.0287 | 42.6226 | 42.6721 |
| 2 | 43.1240 | 43.0848 | 42.9669 | 42.9381 | 42.7048 | 42.7114 |
| 3 | 43.0402 | 43.0705 | 42.9089 | 42.8808 | 42.6683 | 42.6565 |
| 4 | 43.0274 | 43.0161 | 42.8898 | 42.8971 | 42.6845 | 42.6551 |
| 5 | 43.0209 | 43.0103 | 42.8951 | 42.9138 | 42.6611 | 42.6719 |
| 6 | 43.0064 | 42.9552 | 42.8539 | 42.8793 | 42.6463 | 42.6409 |
| 7 | 42.9978 | 42.9511 | 42.8773 | 42.8732 | 42.6416 | 42.6213 |
| 8 | 42.9909 | 42.9429 | 42.8780 | 42.8161 | 42.6694 | 42.6172 |
| 9 | 42.9473 | 42.9487 | 42.8630 | 42.8487 | 42.6335 | 42.6071 |
| 10 | 42.9411 | 42.9105 | 42.8699 | 42.8537 | 42.6785 | 42.5743 |

| BMS |  | WMS |  |
| --- | --- | --- | --- |
| Pitch | 0.0130 | Pitch | 0.0005 |
| Roll | 0.0063 | Roll | 0.0003 |
| Yaw | 0.0013 | Yaw | 0.0009 |
| ICC (1,2) |  |  |  |
| Pitch | 0.9576 |  |  |
| Roll | 0.9444 |  |  |
| Yaw | 0.3154 |  |  |

Minimum angle data

| Cycle | 1^st^ set Pitch | 2^nd^ set Pitch | 1^st^ set Roll | 2^nd^ set Roll | 1^st^ set Yaw | 2^nd^ set Yaw |
| --- | --- | --- | --- | --- | --- | --- |
| 1 | -43.0592 | -43.0630 | -42.9894 | -43.0068 | -42.8273 | -42.8184 |
| 2 | -43.2364 | -43.2515 | -43.2332 | -43.2359 | -42.8180 | -42.8409 |
| 3 | -43.2876 | -43.3338 | -43.2182 | -43.2579 | -42.7969 | -42.7966 |
| 4 | -43.3046 | -43.2984 | -43.2336 | -43.2553 | -42.7905 | -42.7845 |
| 5 | -43.3112 | -43.3002 | -43.2421 | -43.2291 | -42.7461 | -42.8010 |
| 6 | -43.2833 | -43.3100 | -43.1957 | -43.2168 | -42.7486 | -42.7636 |
| 7 | -43.2870 | -43.3265 | -43.2423 | -43.2470 | -42.7360 | -42.8025 |
| 8 | -43.2945 | -43.3246 | -43.2097 | -43.2578 | -42.7340 | -42.7574 |
| 9 | -43.2981 | -43.2964 | -43.2084 | -43.2019 | -42.7358 | -42.7900 |
| 10 | -43.3424 | -43.3611 | -43.2155 | -43.2253 | -42.7244 | -42.7680 |

| BMS |  | WMS |  |
| --- | --- | --- | --- |
| Pitch | 0.0130 | Pitch | 0.0003 |
| Roll | 0.0110 | Roll | 0.0002 |
| Yaw | 0.0017 | Yaw | 0.0006 |
| ICC (1,2) |  |  |  |
| Pitch | 0.9766 |  |  |
| Roll | 0.9754 |  |  |
| Yaw | 0.6125 |  |  |
